# Korean Version of the Swedish Occupational Fatigue Inventory among Construction Workers: Cultural Adaptation and Psychometric Evaluation

**DOI:** 10.3390/ijerph18084302

**Published:** 2021-04-18

**Authors:** Sangeun Lee, Sojeong Seong, Soyeon Park, Jeeyeon Lim, Soyun Hong, Youngshin Cho, Heejung Kim

**Affiliations:** 1 College of Nursing, University of Illinois at Chicago, 845 South Damen Ave., Chicago, IL 60612, USA; slee685@uic.edu; 2 Department of Smart City Engineering, Hanyang University, Ansa 15588, Korea; sjsj601@hanyang.ac.kr (S.S.); soysoyeon@hanyang.ac.kr (S.P.); 3 College of Nursing, Yonsei University, 50-1 Yonsei-ro, Seodaemun-gu, Seoul 03722, Korea; OLIVEJEEYEON@yuhs.ac (J.L.); rnsoyun@gmail.com (S.H.); yshin.cho93@gmail.com (Y.C.); 4 Brain Korea 21 FOUR Project, College of Nursing, Yonsei University, Seoul 03722, Korea; 5 Mo-Im Kim Nursing Research Institute, College of Nursing, Yonsei University, Seoul 03722, Korea

**Keywords:** construction, fatigue, Korean, reliability, validity, workers

## Abstract

The Swedish Occupational Fatigue Inventory (SOFI) has been tested in different languages and populations; thus, there is a need for a culturally adapted Korean version. We evaluated the psychometric properties of a Korean version of the SOFI among construction workers. The SOFI was translated into Korean and reviewed through a back-translation process involving standardized scaling procedures. Its reliability and validity were evaluated with a sample of 193 construction workers using internal consistency, item–subscale correlations, test–retest reliability, and content, construct, and concurrent validity. The Cronbach’s alpha coefficients of the total scale and each subscale were satisfactory. Item–subscale correlations and test–retest reliability were both at acceptable levels. Confirmatory factor analyses revealed that the five-factor model had acceptable model fits corresponding to the structure of the original instrument. However, some modifications were made to improve in the new context from model fit (such as χ^2^(95) = 113.905 (*p* = 0.091), CFI = 0.994, and RMSEA = 0.033, as well as the lowest AIC = 383.905). Correlation analysis showed a significant relationship of SOFI with other fatigue measures in terms of total and subscale scores. Occupational fatigue is one of the important risk factors associated with workers’ health and safety at work. The new translated instrument is a reliable and valid tool for assessing fatigue among Korean construction workers. However, this instrument should be tested extensively in other working populations to devise specific interventions concerning fatigue reduction.

## 1. Introduction

Fatigue is one of the most common symptoms experienced by workers in their daily lives. According to a national fatigue survey of the Korea National Statistical Office in 2018, most married workers experienced time shortages in their daily life and chronic fatigue [1]. In general, fatigue manifests as exhaustion, dysautonomia, and reduced work efficiency; this can result in certain diseases (such as chronic fatigue syndrome, psychosis, depression, stress-related disorder, and autoimmune disease) [2]. Work-related fatigue is highly relevant not only to workers’ health problems but also safety concerns related to preventable death and injury [3,4].

However, it is difficult to measure fatigue. Fatigue is characterized by multidimensional aspects of physical, mental, and functional health, all of which interact with each other [5]. Moreover, fatigue consists of acute and chronic symptoms, which have both subjective and objective features that correspond to situational and individual characteristics. Thus, it is difficult to evaluate the subcomponents of fatigue comprehensively [5]. Diverse types of measurements have been developed, such as self-reporting surveys, electronic devices, and biomarkers that measure cortisol levels, use LED light sources, or apply electrodes to the body [6,7,8]. However, self-reporting surveys are the most frequently used measurement methods in practical or clinical areas in occupational health [5,9] because they are easy to use, save time, and are inexpensive methods compared to the others.

Among the diverse types of self-reporting measures, the Swedish Occupational Fatigue Inventory (SOFI) was developed by Åhsberg and colleagues to evaluate the unique features of momentary fatigue [10]. While other instruments focus on long-term features of fatigue or negative consequences that result from an imbalance between intense workload and delayed recovery [5,11,12], the SOFI focuses on ecological and momentary symptoms here and now to examine instant status or short-termed fatigue symptoms rather than relevant causes or consequences. Several instruments have been devised to measure fatigue symptoms among the general population [13,14,15]. The multidimensional fatigue scale (MFS) [12] and the subjective symptoms of a fatigue test (SSF) [16] are commonly used to identify consistently recurring fatigue [11]. However, because both instruments were devised to assess general populations or subgroups with specific chronic diseases [9,17] (unlike the SOFI), they are insufficient when evaluating immediate fatigue in workers’ daily lives. The instant detection of fatigue is helpful in managing relevant health problems or occupational risk in a timely manner.

This instrument consists of five dimensions: physical exertion, physical discomfort, lack of motivation, sleepiness, and lack of energy [10,18]. The SOFI has been translated into several languages in many countries [19,20,21] and has been tested among diverse occupational groups [10,18,19,20]. This study focused on construction workers who have physically demanding jobs and commonly shift work, both of which are highly associated with high levels of fatigue [20,22]. Considering the very diverse interpretation of fatigue among different individuals, occupational groups, and cultures [10,18,19,20,21], it is necessary to develop a Korean version of the SOFI based on the cultural characteristics of Korea. The aims of this study were to describe the translation and cultural adaptation process of the Korean version of the SOFI, evaluate its psychometric properties by replicating the original model [18], and determine its utility among a sample of Korean construction workers.

## 2. Materials and Methods

### 2.1. Study Design

This study employed a cross-sectional and methodological study to test the psychometric properties of the SOFI.

### 2.2. Participants

Recruited though convenience sampling, 220 workers from one construction site participated in the survey. Inclusion criteria were (1) being aged ≥ 19 years, (2) the ability to understand Korean, (3) at least six months of work experience as a construction worker, and (4) voluntary agreement to participate. Non-Korean or immigrant workers were excluded. Among the 215 respondents who returned completed questionnaires, the data from 193 participants were analyzed after excluding those with missing data. This sample size met the following criteria for a factor analysis: (1) a case–item ratio of nearly 10:1 for an exploratory factor analysis and around 200 cases for a confirmatory factor analysis (CFA) [23]; and (2) that the sample size for a factor analysis is generally about 4–5 times the number of variables, and the number of samples for item analysis is ideally 2–10 times the number of questions [24].

### 2.3. Instrument

#### 2.3.1. SOFI

The SOFI was used to measure self-reported fatigue. It comprises 20 self-reported questions and five dimensional subscales [18]. Each dimension represents different aspects of fatigue: (1) lack of energy, (2) physical exertion, (3) physical discomfort, (4) lack of motivation, and (5) sleepiness. Each item is measured based on a 7-point Likert scale (0 = “not at all,” 3 = “some,” 6 = “to a very high degree”). Total scores ranged from 0 to 120, and higher scores indicate a greater severity of momentary fatigue in the here and now. In the original study [18], internal consistency ranged from 0.81 to 0.92 in total (lack of energy = 0.92, physical exertion = 0.87, physical discomfort = 0.81, lack of motivation = 0.92, and sleepiness = 0.89).

#### 2.3.2. MFS

The Korean version of the MFS is a self-reported questionnaire containing 19 items that assess fatigue type [11,12]. The MFS measures global fatigue, daily dysfunction, and situational fatigue. All items are rated on a seven-point Likert scale (1 = “never” to 7 = “more frequently”). Total scores range from 19 to 133, and higher scores indicate a greater severity of fatigue. The Cronbach’s alpha coefficients were 0.88 in the original study (global fatigue = 0.85, daily dysfunction = 0.79, and situational fatigue = 0.66) and 0.95 in the current study (global fatigue = 0.94, daily dysfunction = 0.90, and situational fatigue = 0.81).

#### 2.3.3. SSF

The SSF was developed by the Japan Industrial Hygiene Association Industrial Fatigue Research Committee [16] and modified by Lee [25] into Korean. The Korean version of the SSF is a measure of fatigue-related symptoms that comprises 30 items measured with a 4-point Likert scale (1 = “not at all” to 4 = “experience always”). The instrument is used to measure physical fatigue (10 items), mental fatigue (10 items), and neuro-sensory fatigue (10 items). Total scores range 30 to 120, and higher scores indicate more severe fatigue. The Cronbach’s alpha coefficients were 0.80 in the original study, 0.95 (physical fatigue = 0.90, mental fatigue = 0.92, and neuro-sensory fatigue = 0.86) in Kim [26], and 0.97 (physical fatigue = 0.91, mental fatigue = 0.92, and neuro-sensory fatigue = 0.93) in the present study.

#### 2.3.4. Socio-Demographic and Health-Related Characteristics

Participants completed a demographics questionnaire comprised of 27 items, including age, height, weight, marital status, education, diagnosed diseases, medication, monthly household income, living arrangement, perceived health status, health screening experience, and working environment (in terms of working type and intensity, among others).

### 2.4. Data Collection Procedure

We collected data between December 2019 and January 2020. Initially, the Korean version of the SOFI was distributed to 220 construction workers at the beginning of their shifts. All participants provided written informed consent and completed the structured questionnaires: SOFI, MFS, SSF, and socio–demographic and health-related information. The test–retest reliabilities of the SOFI, MFS, and SSF were examined 2 weeks after the first investigation. Among the 215 respondents who returned the second questionnaire, the data of 193 participants were included in the data analysis.

### 2.5. Translation and Cultural Adaptation Process

The SOFI was translated using standardized scaling procedures [27]. The researchers received written permission from the original authors to translate the English version of the SOFI into Korean. The accuracy of the translation was verified using a back-translation procedure. Two nursing research assistants who were fluent in Korean and English translated the original English into Korean (version 1). Then, an instrument committee consisting of three nursing professors, a registered nurse, and two construction workers verified the content validity of the preliminary questionnaire to create a Korean version (version 2). After the committee evaluated its completion time, length, feasibility, readability, and comfort through the Delphi method, we conducted a pilot test with 11 Korean construction workers to ensure the feasibility of version 2.

Next, version 2 was back-translated into English by a professional translator and confirmed by another research assistant. Based on the pilot test results, two expert reviewers reassessed the content validity and confirmed its English consistency. Furthermore, additional facial expression and visual analog scales were used at the two extreme values, respectively, and median values were added to clarify the meanings of the ratings (version 3). Version 3 was administered to the 220 construction workers. After completing the data analysis, the instrument committee confirmed the Korean version of the SOFI based on the study findings.

### 2.6. Data Analysis

Data were analyzed using IBM SPSS Statistics Version 25.0 for Windows (IBM, Armonk, NY, USA) and IBM SPSS Amos Version 23.0 (IBM, Armonk, NY, USA). Sample characteristics were analyzed using descriptive statistics. The internal consistency reliability of the SOFI was examined using item–subscale correlations (Spearman’s correlation coefficients) and Cronbach’s alpha. Test–retest reliability was estimated using Pearson’s correlation coefficient. Content validity was calculated as the extent of agreement of the instrument committee, which constituted the content validity index (CVI) [28]. A CFA was performed to evaluate the construct validity of the SOFI. Several indicators were used to assess its model fit including: (1) the normed fit index (NFI) relative fit index (RFI), incremental fit index (IFI), Tucker–Lewis index (TLI), and CFI, all of which should be greater than 0.09; (2) a root mean square error of approximation (0.06 < RMSEA < 0.08); (3) a χ^2^/df ratio (<5.0); and (4) an Akaike information criterion (AIC) indicating that the smaller the AIC, the better the model fit [29].

### 2.7. Ethical Considerations

This study was approved by the affiliated university’s institutional review board (no. Y-2019-0126). All participants provided written informed consent and received a USD 10 gift twice when completing the survey to acknowledge their contribution to this study. Data were anonymized and deidentified with no personal information included, and confidentiality was ensured.

## 3. Results

### 3.1. Participants’ Characteristics

Table 1 displays participants’ demographic characteristics. Participants’ mean age was 47.13 (SD = 11.77) years (range = 23–74 years). Most were men, married, living with family members, and had at least a high school education. The mean working experience on construction sites was 12.90 (SD = 9.55) years. Most worked at least 8 h a day, typically from 9 a.m. to 5 p.m., and most were irregularly employed as a part-time job. Most identified their social economic status as moderate or higher and their health status as fair.

### 3.2. Reliability

#### 3.2.1. Internal Consistency Reliability

Each item within the subscales was reported at a similar level and had few missing data (i.e., less than 2%; Table 2). Cronbach’s alpha of the total SOFI instrument was 0.96 at times 1 and 2 and 0.86 to 0.92 for each subscale at times 1 and 2. Thus, the internal consistency was satisfactory (ranging from 0.70 to 0.90) [30].

#### 3.2.2. Item-to-Subscale Reliability

The items of the SOFI are presented in Table 2. An item-to-subscale analysis was conducted to identify the correlations between each item and its factor. The correlations ranged from 0.71 to 0.82 for physical exertion, 0.68 to 0.76 for lack of motivation, 0.68 to 0.81 for lack of energy, 0.75 to 0.81 for physical discomfort, and 0.74 for 0.88 in sleepiness. The results thus indicated that each item-to-subscale was highly correlated.

#### 3.2.3. Test-Retest Reliability

The test-retest reliability of the SOFI was conducted after two weeks to evaluate the stability of the scale over the time (Table 2). The Pearson’s correlations between the initial (Time 1) and retest (Time 2) responses were 0.82 for the total scale, 0.81 for physical exertion, 0.83 for lack of motivation, 0.82 for lack of energy, 0.82 for physical discomfort, and 0.82 for sleepiness. In addition, the internal consistencies of each of the five subscales at the two-week observations were acceptable.

### 3.3. Validity

#### 3.3.1. Content Validity

Content validity was assessed by examining the differences among the original SOFI, the Korean version of the SOFI, and the back-translated version of the SOFI. It was evaluated twice by the instrument committee. We calculated the item-level CVI (I-CVI) and the scale-level CVI (S-CVI) of the Korean version of the SOFI. The CVI of the SOFI in the pilot and main study ranged from 0.80 to 1.0. I-CVI was 0.99, S-CVI/UA was 0.95, and S-CVI/Ave was 0.99, which were satisfactory [28]. Among the 20 items, the expert panel gave a low score (“somewhat relevant”) to item 7 (sweaty) in the pilot study and item 2 (lack of concern) in the main study (0.80, respectively). Thus, the translation of these two items was revised slightly; however, no items were deleted.

#### 3.3.2. CFA for Construct Validity

**Figure 1:**
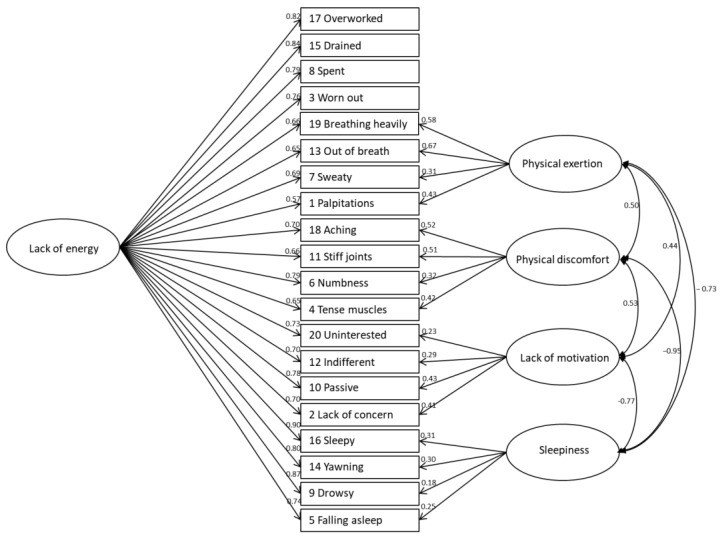
Result of the confirmatory factor analysis (Model 3).

A bifactor CFA was performed to examine the model fit between the hypothesized five-factor model presented in the original study [18] and the data collected from Korean construction workers (Figure 1). The initial model fit (Model 1) with 193 respondents was as follows: χ^2^ (148, *p* < 0.001) = 494.608, CFI = 0.901, NFI = 0.866, RFI = 0.810, IFI = 0.902, TLI = 0.859, and RMSEA = 0.110, 90% CI: 0.100–.121. After the listwise deletion required for modification, Model 2, with data from 179 participants, showed no remarkable differences in model fit indices: χ^2^ (148, *p* < 0.001) = 499.574, CFI = 0.864, NFI = 0.858, RFI = 0.818, IFI = 0.896, TLI = 0.864, and RMSEA = 0.116, 90% CI: 0.104–0.127. Some minor modifications were required to allow for the correlated errors of measured variables (Model 3) that displayed the best model fit, and they were determined as the variables for the final model (Table 3).

#### 3.3.3. Concurrent Validity

Concerning concurrent validity, Table 4 shows the correlation coefficients among the SOFI, MFS, and SSF total and subscale scores. All correlations were moderate and positive (all *p* < 0.01).

## 4. Discussion

We developed a Korean version of the SOFI and examined its reliability and validity in a sample of construction workers. Our findings support the conclusion that the Korean version of the SOFI is a reliable and valid instrument to evaluate momentary work-related fatigue among construction workers. Similar to the original SOFI, five factors are recommended; however, there is a possible concern of underreporting or incorrect perception of each item in Korean version that possibly suggests an inaccuracy in the modified model.

The Korean version of the SOFI displayed satisfactory internal consistency reliability, item–subscale reliability, and test–retest reliability, which are valuable metrics for newly developed instruments [31]. Our internal consistency coefficients indicated high levels of reliability for all items, which was similar to those of the Chinese (25 items) [20] and Portuguese (20 items) [21] versions and somewhat higher than that of the Spanish version (15 items) [19]. The item–subscale reliability was moderate to high and test–retest reliability was strong, similar to the original SOFI [18]. The reliability results are expected since all participants worked in the same occupation. Homogeneity of the sample may increase reliability because the respondents experience similar fatigue symptoms due to comparable tasks or working environments [20,21], as the original study discussed [18].

Our study findings demonstrate an acceptable fit to the hypothesized five-factor model with minor modification. Similar to previous studies [18,20], “lack of energy” was a general latent factor and the other four constructs (physical exertion, physical discomfort, lack of motivation, and sleepiness) were separately identified in a similar manner to the original SOFI [18]. Compared to the original model [18], the factor loadings of each factor were somewhat different: (1) there were higher factor loadings of each measured item for the lack of energy in our study and (2) there were much smaller factor loadings of four other factors, specifically for lack of motivation and sleepiness. In the original study [18], the correlation between factors for lack of energy varied between 0.28 and 0.86, whereas this study showed factor loading ranging from 0.57 to 0.90. For the other four latent factors, the values ranged between 0.43 and 0.90 in the original model [18]. On the contrary, each factor reported factor loading ranging from 0.18 to 0.67, especially those of lack of motivation and sleepiness (which scored lower than 0.43 in this study). This difference may result from our choice to perform a bifactor CFA instead of a hierarchical CFA. Considering the higher correlations among factors in our study than those in the original model [18], we may need to choose the hierarchical CFA to enhance model fit to the data.

Moreover, the highest loaded items differed from the findings reported in the other SOFI translation studies [19,20]. Moreover, some items such as items 9 (drowsy), 12 (indifferent), and 20 (uninterested) displayed low loading values as compared to those present in previous studies [19,20,21]. In addition, our findings suggest a re-evaluation of the validity of lack of motivation and sleepiness by re-assessing “uninterested” and “indifferent” for lack of motivation and “drowsy” and “falling asleep” for sleepiness. In general, this discrepancy may result from cultural effects or language differences concerning the perception, interpretation, and expression of work-related fatigue [20]. These concepts are difficult to distinguish; thus, the possible suppression of these items may occur [19].

Concerning concurrent validity, the total scale and all subscales were positively and moderately associated with other fatigue measures (MFS and SSF). In addition, a strong correlation was reported between the SOFI subscales “lack of energy” and “sleepiness” and the MFS. In addition, physical functioning in the SSF was highly correlated with “lack of energy” and “sleepiness” in the SOFI. The SOFI may be more sensitive at measuring the physical aspects of fatigue such as “physical functioning” and “lack of energy.” However, “situational fatigue” in the MSF and “neuro-sensory fatigue” were less sensitive to measure the symptoms of fatigue with construction workers. In general, fatigue is comprised of multidimensional features and is easily influenced by task type and workload [21]. Our sample of construction workers work physically demanding jobs and are vulnerable to relevant dysfunction and fatigue [32]. In previous studies, the SOFI was used to assess physical symptoms, especially among patients with chronic diseases or specific workers [19,21]. However, Åhsberg et al. [18] found that the SOFI could also be used to assess perceived mental aspects of fatigue in people with mental health problems. Thus, further studies should include diverse study participants considering each construct (physical, mental, and functional aspects).

Fatigue is a consequence of interaction between physical and psychological aspects perceived by each individual, and it is also greatly influenced by culture [33]. The evaluation of momentary fatigue is important to promote the health and safety of construction workers and occupational healthcare providers, especially considering the culturally sensitive perception of fatigue [20]. The differences in the factor structure of the SOFI between South Korea and other countries may be attributed to cultural differences concerning the perception of fatigue, specific samples, and the newly translated measure. Therefore, some items will differ between Korea and other countries; for example, “lack of concern” vs. “passive,” “stiff joint” vs. “aching,” “indifferent” vs. “uninterested,” and “drowsy” vs. “drained.” Thus, the enhanced standardized development of the instrument, cultural adaptation, and further modifications are critical [27].

This study followed the guidelines of Netemeyer et al. [27]. We confirmed the final version after contextual evaluations with clinical and professional experts and construction workers. However, some items were hard to understand owing to the relatively lower levels of education and health literacy among construction workers. Some researchers emphasized that participants’ education level should be considered when developing health-related instruments [34,35]. In the original instrument, two extreme values of a 7-point Likert scale were provided (0 = “not at all,” 6 = “to a very high degree”). Compared to a previous study [20], we made some revisions considering participants’ relatively low levels of education. Thus, we added 3 (“somewhat”) at the mid-point. In addition, we added facial expression symbols to assist participants. This modification would be helpful to apply SOFI to low-educated individuals with little experience regarding health-related survey (such as older adults or immigrants).

In further studies, we may consider shortening the items, similar to the 15-item Spanish version [19]. We did not reduce the number of items or move a specific item to another construct; however, we made few modifications by allowing for correlations among measurement errors statistically. This means that the unmeasured areas of each of the items were closely related to each other; thus, underreporting might be a possible concern. As some modification is required, as suggested in the original SOFI study [18], we may consider the further modifications. However, before shortening the 20-item SOFI, we need to test the current form with different groups. It is necessary to consider workload (such as task demand, performance, effort, or frustration levels) [18,19]. Previous studies explained that poor model fit may result from the homogeneity of the tested sample, when compared to the initial study samples with 14 different professions [10,18]. This problem also occurred in the Spanish version of SOFI that exclusively included nurses or sedentary workers [19,20]. A sample consisting of workers from the same occupation or with similar work types may increase poor model fit [18,19,20], similar to our study’s inclusion of only construction workers. Thus, future studies should include more heterogeneous samples considering assigned tasks and occupation. In addition, further studies should be conducted with female and non-Korean foreign workers because they may have different experiences from the majority of Korean men workers.

There were several limitations to this study. First, our Korean translated SOFI cannot determine the pathogenic levels of fatigue that are required for close attention and medical treatment. For example, the Fatigue Severity Scale and its specific cutoff have been used to screen vulnerable groups for long-term fatigue requiring medical care [36]. Since the SOFI is useful to detect momentary workplace fatigue, developing a cutoff is required to determine the severity and the right time to provide effective interventions to decrease workers’ fatigue. Secondly, some items were similar; thus, duplicated factor loadings may have occurred owing to fatigue characteristics or misunderstandings owing to Korean cultural differences. It is necessary to replicate this study with a larger sample and among diverse occupations [20,37,38] with different demands or socio-demographic characteristics, such as women or non-Korean foreign workers [18,19,20]. Thirdly, we collected data from 6–7 a.m.; however, fatigue may change within the day owing to participants’ circadian rhythm [39,40]. That is to say, fatigue could be affected by the measurement time such as morning vs. afternoon or shift worker vs. 9–5 worker (because fatigue is a time-dependent variable) [39,41]. Fourthly, this study partially examined the responsiveness of Korean construction workers at the pilot test. Since responsiveness is the one of the useful indexes to reflex the extent of the changes [42], it might be an effective indication that can detect distinction of the information for the instrument when changes in it have occurred. The next study should examine in-depth responsiveness to enhance users’ willingness to use SOFI considering the importance of a subjective, accurate, and timely report of fatigue. Lastly, it is important to distinguish fatigue from other similar conditions such as daytime sleepiness, depression, anxiety, occupation circumstances, or sex because fatigue is the most commonly reported symptom in primary care settings and population [36]. It is necessary to assess fatigue several times within a day and assess workers who complete diverse tasks and have similar health problems. In addition, it is possible to use both subjective and objective measurements simultaneously [20].

## 5. Conclusions

This Korean version of the SOFI is a valid and reliable instrument for evaluating momentary fatigue among Korean construction workers considering its cultural adaptation to Korean. Our findings also enhance the cultural understanding of momentary fatigue and contribute to the development of work-related fatigue reduction programs in their working environment. Effectively assessing construction workers’ fatigue in a timely manner may help in the reduction of fatigue-related health problems and safety concerns. For future research, it is necessary to further examine diagnostic validity considering a cutoff to identify severe fatigue groups and ensure the expansion of the use of SOFI with more diverse types of workers.

## Figures and Tables

**Table 1:** Characteristics of the participants (N = 193).

Characteristics	*n* (%)
Gender	
Men	163 (84.5)
Women	30 (15.5)
Marital status	
Never married	42 (21.8)
Married	141 (73.1)
Divorced or separated	7 (3.6)
Widowed or others	3 (1.5)
Living arrangement	
Living alone	19 (9.9)
Living with family members	151 (78.2)
Living with nonfamily members	23 (11.9)
Education	
Middle school or under	24 (13.5)
High school	71 (36.8)
College or above	96 (49.7)
Working hour of a day (h)	
8 h or above	134 (69.4)
Less than 8 h	59 (30.6)
Occupational status	
Regular employment as a full-time job	65 (33.7)
Irregular employment as a part-time job	126 (65.3)
Work shift	
9 a.m. to 5 p.m.	167 (86.5)
Irregular shift including night shift	26 (13.5)
Social economic status	
Above the moderate level	27 (14.0)
Moderate	84 (43.5)
Below the moderate level	82 (42.5)
Perceived health status	
Excellent	3 (1.6)
Good	29 (15.0)
Fair	117 (60.6)
Poor	42 (21.8)

**Table 2:** Item subscale correlations and the missing numbers and their percentages for the 20-item Swedish Occupational Fatigue Inventory (N = 193).

	Subscale and Item	M (SD)	Missing Number (%)	Item–Subscale Correlation	Test–Retest Reliability	Subscale’s Cronbach Alpha	
						Time 1	Time 2
	SOFI_total				0.82	0.96	0.96
Physical Exertion					0.81	0.86	0.89
1	Palpitations	1.38 (1.40)	0 (0)	0.71			
7	Sweaty	1.46 (1.50)	1 (0.5)	0.72			
13	Out of breath	1.42 (1.54)	1 (0.5)	0.82			
19	Breathing heavily	1.27 (1.48)	0 (0)	0.77			
Lack of Motivation					0.83	0.87	0.87
2	Lack of concern	1.72 (1.54)	1 (0.5)	0.73			
10	Passive	1.99 (1.54)	2 (1.0)	0.76			
12	Indifferent	2.16 (1.57)	2 (1.0)	0.75			
20	Uninterested	2.01 (1.57)	0 (0)	0.68			
Lack of Energy					0.82	0.87	0.89
3	Worn out	2.26 (1.64)	2 (1.0)	0.68			
8	Spent	1.79 (1.51)	1 (0.5)	0.74			
15	Drained	2.08 (1.62)	0 (0)	0.81			
17	Overworked	2.45 (1.68)	1 (0.5)	0.76			
Physical Discomfort					0.82	0.88	0.90
4	Tense muscles	2.03 (1.58)	0 (0)	0.75			
6	Numbness	1.83 (1.48)	2 (1.0)	0.75			
11	Stiff joints	1.91 (1.62)	1 (0.5)	0.81			
18	Aching	1.77 (1.58)	0 (0)	0.78			
Sleepiness					0.82	0.92	0.92
5	Falling asleep	2.42 (1.59)	0 (0)	0.74			
9	Drowsy	2.40 (1.48)	2 (1.0)	0.80			
14	Yawning	2.58 (1.56)	0 (0)	0.81			
16	Sleepy	2.54 (1.71)	2 (1.0)	0.88			

**Table 3:** Model-fit indices among competing models.

Characteristics		χ^2^ (df, *p*)	NFI	RFI	IFI	TLI	CFI	RMSEA (90% CI)	AIC
Model 1	Original model	494.068 (148, <0.001)	0.866	0.810	0.902	0.859	0.901	0.110 (0.100, 0.121)	658.068
Model 2	Listwise deletion model	499.574 (148, <0.001)	0.858	0.818	0.896	0.864	0.864	0.116 (0.104, 0.127)	663.574
Model 3	Finalized model	113.905 (95, 0.091)	0.968	0.935	0.994	0.989	0.994	0.033 (<0.001, 0.054)	383.905

Notes: NFI = Normed fit index; RFI = Relative fit index; IFI = Incremental fit index; TLI = Tucker–Lewis index; CFI = Comparative fit index; RMSEA = Root mean square error of approximation; AIC = Akaike information criterion.

**Table 4:** Correlations among subscale and total score of SOFI, MFS, and SSF (N = 193).

	Variables	1	2	3	4	5	6	7	8	9	10	11	12	13
1	SOFI_total	1												
2	SOFI_PE	0.847 **	1											
3	SOFI_LM	0.916 **	0.759 **	1										
4	SOFI_LE	0.935 **	0.726 **	0.797 **	1									
5	SOFI_PD	0.886 **	0.756 **	0.815 **	0.756 **	1								
6	SOFI_S	0.828 **	0.521 **	0.678 **	0.837 **	0.589 **	1							
7	MFS_total	0.594 **	0.382 **	0.523 **	0.625 **	0.487 **	0.590 **	1						
8	MFS_GF	0.546 **	0.358 **	0.501 **	0.570 **	0.458 **	0.511 **	0.924 **	1					
9	MFS_DD	0.562 **	0.352 **	0.501 **	0.582 **	0.446 **	0.583 **	0.907 **	0.749 **	1				
10	MFS_SF	0.452 **	0.283 **	0.357 **	0.500 **	0.369 **	0.472 **	0.815 **	0.617 **	0.666 **	1			
11	SSF_total	0.591 **	0.484 **	0.516 **	0.580 **	0.492 **	0.530 **	0.484 **	0.423 **	0.494 **	0.353 **	1		
12	SSF_PF	0.615 **	0.482 **	0.534 **	0.587 **	0.505 **	0.597 **	0.516 **	0.411 **	0.542 **	0.423 **	0.912 **	1	
13	SSF_MF	0.529 **	0.411 **	0.478 **	0.534 **	0.406 **	0.498 **	0.444 **	0.398 **	0.462 **	0.300 **	0.926 **	0.798 **	1
14	SSF_NSF	0.486 **	0.437 **	0.413 **	0.478 **	0.445 **	0.373 **	0.377 **	0.354 **	0.362 **	0.257 **	0.907 **	0.724 **	0.748 **

Notes: SOFI = Swedish Occupational Fatigue Inventory; PE = Physical exertion; LM = Lack of motivation; LE = Lack of energy; PD = Physical discomfort; S = Sleepiness; MFS = Multidimensional Fatigue Scale; GF = Global fatigue; DD = Daily dysfunction; SF = Situational fatigue; SSF = Subjective Symptoms of Fatigue; PF = Physical fatigue; MF = Mental fatigue; NSF = Neuro-sensory fatigue. ** *p* < 0.01.

## Data Availability

The data are not publicly available due to protection of subjects’ privacy and confidentiality. The data presented in this study are available on request from the corresponding author as well as the Korean version of SOFI.
